# Conformational plasticity and truncational effects on bovine lactoferricin: structural determinants of enhanced antimicrobial activity

**DOI:** 10.3389/fcimb.2026.1888171

**Published:** 2026-07-10

**Authors:** Jie Pei, Lin Xiong, Qianyun Ge, Xiaoyun Wu, Min Chu, Pengjia Bao, Xian Guo

**Affiliations:** 1 Key Laboratory of Yak Breeding in Gansu Province, Lanzhou Institute of Husbandry and Pharmaceutical Sciences, Chinese Academy of Agricultural Sciences, Lanzhou, Gansu, China; 2 Key Laboratory of Animal Genetics and Breeding on Tibetan Plateau, Ministry of Agriculture and Rural Affairs, Lanzhou, Gansu, China

**Keywords:** antibacterial activity, antimicrobial peptide, lactoferricin, secondary structure, truncated peptide

## Abstract

**Background:**

The rapid emergence of multidrug-resistant bacterial pathogens has created an urgent demand for alternative antimicrobial agents. Bovine lactoferricin (Lfcin B), a cationic antimicrobial peptide derived from bovine lactoferrin, exhibits potent broad-spectrum antibacterial activity. Although truncated derivatives of Lfcin B retain partial antimicrobial effects, their efficacy relative to full-length Lfcin B and the structural mechanisms governing their function remain poorly understood.

**Methods:**

Full-length Lfcin B and three truncated variants (Lfcin B15, Lfcin B9, and Lfcin B6) were synthesized using solid-phase peptide synthesis and characterized by RP-HPLC and MALDI-TOF-MS. Circular dichroism spectroscopy was employed to evaluate secondary structural changes under different ionic and hydrophobic environments. Tertiary structures were predicted using AlphaFold3. Antibacterial activities were assessed against multidrug-resistant Gram-negative and Gram-positive pathogens, including *Escherichia coli*, *Klebsiella pneumoniae*, *Pseudomonas aeruginosa*, *Salmonella typhimurium*, *Salmonella gallinarum*, *Shigella flexneri*, *Staphylococcus aureus*, and *Trueperella pyogenes*, using MIC, MBC, and agar diffusion assays.

**Results:**

Circular dichroism spectroscopy revealed that ionic strength and hydrophobic environments modulate the secondary structures of the peptides, with increased ionic strength consistently reducing random coil ratios across all variants. Structural stability progressively diminished with peptide truncation, as shorter variants exhibited less conformational complexity. AlphaFold3-predicted tertiary structures identified two distinct conformations for full-length Lfcin B, an α-helix-rich state and a β-sheet-dominant topology, whereas truncated variants adopted simpler structural ensembles, primarily α-helical or random coil conformations. Antibacterial activity decreased markedly with peptide truncation. Lfcin B demonstrated the strongest and broadest-spectrum antibacterial activity, showing substantially lower MIC and MBC values and larger inhibition zones than truncated peptides. In contrast, Lfcin B6 exhibited only limited activity against *T. pyogenes*. Structural analyses indicated that the intact sequence and intramolecular disulfide bond of Lfcin B are essential for maintaining conformational stability, membrane interaction capacity, and antibacterial potency.

**Conclusions:**

The antibacterial efficacy of bovine Lfcin B is strongly associated with its full-length sequence, conformational adaptability, and disulfide bond-mediated structural stability. Progressive truncation compromises structural plasticity and significantly attenuates antimicrobial activity. These findings support Lfcin B as a promising structural scaffold for the development of next-generation therapeutics against antibiotic-resistant bacterial infections.

## 1 Introduction

Bacterial pathogens remain a leading global cause of human morbidity and mortality (Pei et al., 2025). The discovery of antibiotics revolutionized medicine by providing rapid, effective treatment for bacterial infections, significantly improving health outcomes and extending life expectancy (Cook and Wright, 2022). However, widespread overuse and misuse of antibiotics have accelerated the emergence of antibiotic-resistant strains, which has critically diminished the efficacy of major antibiotic classes (Abbas et al., 2024). A particularly alarming consequence is the rise of multidrug-resistant (MDR) pathogens that evade multiple antibiotic families (Bharadwaj et al., 2022). Foremost among these are the ESKAPE pathogens, including *Enterococcus faecium*, *Staphylococcus aureus* (*S. aureus*), *Klebsiella pneumoniae* (*K. pneumoniae*), *Acinetobacter baumannii*, *Pseudomonas aeruginosa* (*P. aeruginosa*), and *Enterobacter* spp. These pathogens are prioritized by the WHO as requiring urgent therapeutic solutions. Within clinical environments, ESKAPE pathogens frequently demonstrate a rapid capacity to adapt to antibiotic exposure, acquiring resistance traits that compromises clinical efficacy (Daruka et al., 2025). This persistent challenge underscores the critical need for innovative strategies, emphasizing novel antimicrobial agents and resistance-mitigation approaches (Brüssow, 2024).

Antimicrobial peptides (AMPs) are diverse naturally occurring molecules and constitute a critical component of the innate immune system. They serve as the first line of defense against broad-spectrum pathogens (Zhou et al., 2022). Unlike traditional antibiotics, AMPs employ multimodal mechanisms of action initiated by electrostatic interactions with anionic microbial membranes, which triggers membrane disruption via pore formation, carpeting, or detergent-like lysis (Islam et al., 2023). Subsequent intracellular penetration enables targeted inhibition of vital processes, including protein/DNA synthesis, enzymatic functions, and cell division, through interactions with anionic biomolecules (Brogden, 2005; Le et al., 2017; K R et al., 2024; Oliveira Júnior et al., 2025). Collectively, these multifaceted mechanisms significantly impede bacterial resistance development through genetic mutations (Lazzaro et al., 2020). Owing to their broad-spectrum efficacy, multifunctionality, and reduced resistance propensity, AMPs represent promising candidates for novel therapeutic agents against antibiotic-resistant infections (Maria et al., 2020). Despite their therapeutic promise, the clinical translation of AMPs faces significant hurdles, including high manufacturing costs associated with large-scale solid-phase peptide synthesis, rapid proteolytic degradation by host proteases *in vivo*, and potential systemic toxicities such as hemolytic activity (Dijksteel et al., 2021; Lai et al., 2022; Zheng et al., 2025). Addressing these limitations via structural minimization and conformational stabilization represent a central objective in next-generation AMP design.

Lactoferricin (Lfcin), a highly potent AMP, is derived from the proteolytic cleavage of lactoferrin (LF) and represents the core antimicrobial domain of LF (Wakabayashi et al., 2003). Among mammalian Lfcins, the bovine Lfcin (Lfcin B) demonstrates particularly strong antibacterial activity (Vorland et al., 1998; Sebastien et al., 2004). Lfcin B is a 25-amino acid peptide corresponding to residues 17–41 of mature bovine LF (Bellamy et al., 1992). This sequence contains eight positively charged and ten hydrophobic residues, which confer amphipathic properties essential for its potent antibacterial action against diverse pathogenic bacteria (Hans J et al., 2002; Sun et al., 2018). Lfcin B exhibits unique molecular characteristics and potent antimicrobial activity. Therefore, it serves as a promising candidate for structure-based design strategies. These strategies aim to develop novel therapeutic agents against infectious pathogens (Svendsen et al., 2019).

The antibacterial function of Lfcin B primarily depends on its abilities to permeabilize cell membranes and bind cellular targets (Libby et al., 2024). Enhanced membrane permeability likely facilitates more effective disruption of the plasma membrane, thereby increasing antibacterial efficacy. Previous studies have indicated that some truncated Lfcin B variants retain substantial antibacterial activity, whereas others exhibit reduced potency (Wakabayashi et al., 1999; Svenson et al., 2010; Huertas et al., 2017). However, critical knowledge gaps remain: the minimal truncation length required to preserve the antibacterial activity has not been established, and the precise structure-function relationships governing the activity of these truncated variants are poorly understood.

**Figure 1:**
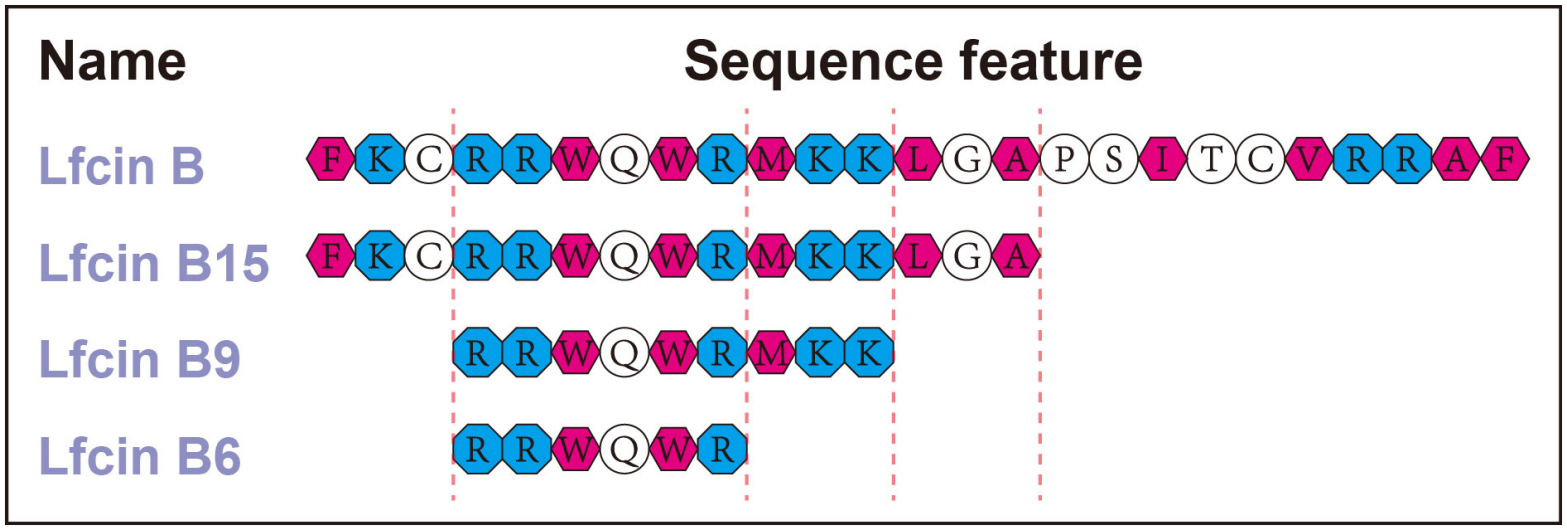
Schematic representation of Lfcin B and its truncated variants. Key structural features are illustrated, with basic amino acid residues depicted as cyan octagons and hydrophobic amino acid residues represented by magenta hexagons. Color and shape coding are employed to enhance visual distinction between residue classes.

To investigate the relationship between peptide length, structural dynamics, and antimicrobial function, we synthesized and characterized Lfcin B and three truncated variants: Lfcin B15, Lfcin B9, and Lfcin B6 (Schematic structures depicted in **Figure 1**). Secondary structure conformational changes were probed using Circular Dichroism (CD) spectroscopy under systematically varied ionic strength and hydrophobic conditions. Concurrently, the antibacterial activities of these peptides were evaluated against a panel of clinically significant, multidrug-resistant bacterial pathogens. This panel included *Escherichia coli* (*E. coli*), *K. pneumoniae*, *P. aeruginosa*, *Salmonella typhimurium* (*S. typhimurium*), *Salmonella gallinarum* (*S. gallinarum*), *Shigella flexneri* (*S. flexneri*), *S. aureus*, and *Trueperella pyogenes* (*T. pyogenes*), selected for their global public health threat due to extensive antibiotic resistance. This study directly addresses the length-dependent conformational dynamics of Lfcin B and its functional consequences on antimicrobial efficacy. Our findings contribute to understanding the structure-activity relationships of AMPs and inform the potential development of Lfcin B-inspired therapeutics.

## 2 Methods

### 2.1 Reagents and chemicals

Mueller-Hinton agar (MHA), Mueller-Hinton broth (MHB), *E. coli* ATCC 25922, *K. pneumoniae* ATCC 4352, *P. aeruginosa* ATCC 27853, *S. typhimurium* ATCC 14028, *S. flexneri* ATCC 12022, *S. aureus* ATCC 25923, and *T. pyogenes* ATCC 19411 were procured from the American Type Culture Collection (ATCC, VA, USA). *S. gallinarum* was isolated from liver tissues of chickens that had died from naturally occurring infections on commercial farms and identified by biochemical tests, serotyping, and molecular methods (Sannat et al., 2017). A *T. pyogenes* isolate was obtained from mastitic cow milk and identified using established methods (Hijazin et al., 2011; Pei et al., 2020). No live animals were used or experimentally handled in this study. The use of animal-derived samples complied with the Animal Administration and Ethics Committee of Lanzhou Institute of Husbandry and Pharmaceutical Sciences, Chinese Academy of Agricultural Sciences, and complied with relevant guidelines and regulations. Chemical reagents, including 1,2-ethanedithiol (EDT), 4-methylpiperidine, pyridine, N,N-diisopropylethylamine, ninhydrin, and triisopropylsilane (TIPS), were sourced from Sigma-Aldrich (MO, USA). Fmoc-protected amino acids, N,N-dicyclohexylcarbodiimide (DCC), rink amide resin, and 6-chloro-1-hydroxy-benzotriazole (6-Cl-HOBt) were obtained from AAPPTec (KY, USA). Solvents and other materials, including absolute ethanol, acetonitrile (ACN), dichloromethane, diethyl ether, isopropyl alcohol, methanol, N,N-dimethylformamide (DMF), and trifluoroacetic acid (TFA), were purchased from Honeywell-Burdick & Jackson (MI, USA). All reagents were used without further purification.

### 2.2 Peptide synthesis

The peptides were synthesized manually via solid-phase peptide synthesis (SPPS) using the Fmoc/tBu strategy (Remuzgo et al., 2014). Rink amide resin (0.46 meq/g loading capacity) served as the solid support. The synthesis protocol proceeded as follows: (a) The resin-bound Fmoc group was removed by treatment with 20% (v/v) 4-methylpiperidine in DMF. (b) Fmoc-protected amino acids (0.21 mmol) were preactivated with DCC (0.20 mmol) and 6-Cl-HOBt (0.21 mmol) in DMF at 25 °C, followed by coupling to the resin for 60 min under constant mechanical agitation. The coupling efficiency was monitored at each step using the quantitative Kaiser test, routinely exceeding 98.5%. (c) The peptide-resin complex was treated with a cleavage cocktail composed of TFA/H_2_O/TIPS/EDT (93:2:2.5:2.5, v/v) at a ratio of 10 mL/g resin for 2.5 h at room temperature (25 °C) to simultaneously liberate the peptide from the resin and remove side-chain protecting groups. (d) Crude peptides were precipitated with cold diethyl ether, dried under ambient conditions.

### 2.3 Peptide purification and identification

The crude peptides were purified via solid-phase extraction using a Supelclea LC-18 column. After loading the peptides onto the column, elution was performed with a gradient of solvent B. The collected fractions were subsequently analyzed by reversed-phase high-performance liquid chromatography (RP-HPLC) and mass spectrometry (MS) (Lima et al., 2020). For RP-HPLC analysis, 20 μL of the crude peptide stock solution (1 mg/mL) was injected into a C18 column (Kromasil, 5 μm particle size, 4.6 × 150 mm dimensions) using an Agilent 1200 liquid chromatograph (Agilent Technologies, NE, USA). Separation was achieved with a linear gradient from 20% to 50% solvent B (0.1% TFA in ACN) in solvent A (0.1% TFA in water) over 25 minutes. The eluted peptides were monitored at 220 nm with a flow rate of 1.0 mL/min and a column temperature of 25 °C. Only synthetic peptide fractions exhibiting a minimum purity threshold of ≥ 95%, as validated by analytical RP-HPLC, were accepted for downstream physical and biological evaluations. Matrix-assisted laser desorption/ionization time-of-flight mass spectrometry (MALDI-TOF-MS) analysis was conducted on an Ultraflex III TOF/TOF mass spectrometer (Bruker Daltonics, Bremen, Germany) in reflectron mode. Samples were spotted onto an MTP384 polished steel target (Bruker Daltonics) using 2,5-dihydroxybenzoic acid or sinapinic acid as the matrix. Spectra were acquired with 500 laser shots at 25–30% laser power.

### 2.4 Circular dichroism spectroscopic analysis

CD spectra of the synthetic peptides were acquired using a Chirascan spectropolarimeter (Applied Photophysics Ltd, UK). Each purified peptide was dissolved in one of the following solvents to a final concentration of 0.16 mg/mL: ultrapure water (H_2_O), PBS (5.8 mmol/L phosphate, 58.3 mmol/L NaCl, pH 7.0), PBS containing 0.56 mmol/L sodium dodecyl sulfate (SDS), or PBS containing 8.33 mmol/L SDS. The SDS concentrations were strategically chosen based on its critical micelle concentration (CMC), which occurs at ~ 8.0 mM. Thus, 0.56 mM SDS establishes a sub-micellar environment dominated by monomeric surfactant interactions, whereas 8.33 mM SDS creates a supramicellar, membrane-mimetic matrix featuring fully assembled hydrophobic-anionic micelles. Peptide aggregation was monitored indirectly prior to spectral analysis by measuring solution turbidity (OD600) and enforcing a standard filtration step (0.22 μm). No visible or spectrophotometric signs of aggregation were detected at the working concentration of 0.16 mg/mL. Maintaining a uniform mass concentration across variants ensures an equivalent concentration of peptide backbone amide bonds within the optical pathlength, which directly governs far-UV absorbance. This standardized approach allows for direct comparison of structural conformations independent of variations in molar concentration resulting from different molecular weights. Measurements were performed at 25 °C in a quartz cuvette with a 0.5 mm pathlength. Far-UV spectra were recorded across a wavelength range of 180~260 nm using a 1.0 nm step size, 1.0 nm bandwidth, and 0.5 s response time. For each sample, three consecutive scans were averaged to enhance signal-to-noise ratio. A baseline spectrum of the corresponding solvent was acquired under identical conditions and subtracted from the peptide spectra prior to analysis.

The mean residue ellipticity (***MRE***) was calculated using the formula:


MRE = (θ × 0.1 × MRW)/cl

where ***θ*** is the measured ellipticity (in millidegrees), ***c*** is the peptide concentration (in mg/mL), ***l*** is the cuvette pathlength (in cm), and ***MRW*** (mean residue weight) is defined as:


MRW = MW/(n − 1)

Here, ***MW*** represents the molecular weight of the peptide, and ***n*** is the total number of amino acid residues.

### 2.5 Quantitative secondary structure analysis

Quantitative secondary structure analysis of the smoothed, baseline-subtracted CD spectral data was executed via neural network deconvolution using the CDNN software suite (Applied Photophysics Ltd.). Deconvolution algorithms utilized the complex neural network based on 33 reference base-spectra, taking into account experimental inputs of mean residue weight, exact pathlength, and mass concentrations to estimate fractional secondary structural components (Pei et al., 2025).

### 2.6 Tertiary structure prediction via AlphaFold3

The three-dimensional structures of Lfcin B and its truncated peptides were predicted using AlphaFold3 (v3.0.0, DeepMind), a deep learning framework that combines evolutionary covariation patterns, physicochemical constraints, and geometric neural networks to model protein folding dynamics (Abramson et al., 2024). Monomeric peptide sequences in FASTA format were submitted to the AlphaFold3 Server (https://golgi.sandbox.google.com/) with the following parameters: molecular entity type designated as “protein”, oligomerization state restricted to a single polypeptide chain (copy_number = 1), and post-translational modifications explicitly disabled to focus on intrinsic folding behavior. To account for conformational diversity, eight independent structural models per target were generated using distinct pseudorandom seed values, enabling exploration of alternative folding pathways within the energy landscape. Model selection was guided by a hierarchical evaluation of structural confidence metrics: local residue-level reliability was assessed via predicted Local Distance Difference Test scores, prioritizing core regions with thresholds >80 to ensure high-confidence rigid domains; inter-domain interaction fidelity was evaluated using interface Prediction-TM scores (>0.7) to validate residue packing and interfacial alignment; and steric/topological integrity was optimized by rejecting models with atomic clashes exceeding tolerance thresholds or violating conserved fold topologies.

To validate the computational predictions, the conformational ensembles were cross-validated against two experimentally resolved structures: (1) the solution-state NMR structure (PDB: 1LFC), which captures the peptide’s dynamic behavior in aqueous buffer, and (2) the X-ray crystallography structure of full-length lactoferrin (PDB: 1BLF), revealing the intramolecularly constrained conformation adopted by Lfcin B within the native protein.

### 2.7 Antibacterial activity assays

Since no CLSI (Clinical and Laboratory Standards Institute) criteria define minimum inhibitory concentration (MIC) breakpoints for AMPs, antibacterial assays were performed using a modified broth dilution method as previously described (Wiegand et al., 2008). The broth microdilution assay was employed to determine both MIC and minimum bactericidal concentration (MBC). Each bacterial strain was cultured in MHB and incubated at 37 °C with shaking (200 rpm) for 12~15 h until reaching an optical density (OD_620_) of 0.15–0.30. A 75 μL aliquot of peptide solution (4 mg/mL) was dispensed into the first column of a 96-well polypropylene microtiter plate (Costar 3790, Corning Inc., USA). Two-fold serial dilutions were prepared across columns 1~10 using 2×AABSA solution (0.2 μL/mL acetic acid, 4 mg/mL BSA). An equal volume (75 μL) of bacterial inoculum (1 × 10^6^ CFU/mL) was then added to each well, achieving a final bacterial concentration of 5 × 10^5^ CFU/mL and final peptide concentrations of 1000, 500, 250, 125, 62.5, 31.25, 15.63, 7.81, 3.91, and 1.95 μg/mL. The total reaction volume in each well was 150 μL. The plates were incubated at 37 °C with shaking (200 rpm) for 24 h. Bacterial growth was quantified by measuring OD_620_ using an Asys Expert Plus microplate reader.

MIC_50_, MIC_90_, and MBC were determined using a standardized bacteriological approach. A 100 μL aliquot from each well was subjected to ten-fold serial dilution, and dilutions were spread onto MHA plates. Following incubation at 37 °C for 24 h, plates containing 30~300 colonies were selected to calculate the original CFU/mL, which was used to derive MIC_50_, MIC_90_, and MBC values. MIC_50_ was defined as the minimum peptide concentration required to inhibit ≥50% of bacterial growth relative to peptide-free control plates, while MIC_90_ represented the minimum concentration achieving ≥90% growth inhibition. MBC was defined as the lowest peptide concentration resulting in a ≥99.9% reduction in bacterial counts compared to untreated controls (Wiegand et al., 2008; Lewis, 2010). To ensure assay validity and experimental reproducibility, three independent sets of controls were strictly maintained on every 96-well microtiter plate during MIC and MBC determinations: (i) a positive control consisting of standard reference antibiotics, Ampicillin for Gram-negative targets and Gentamicin for Gram-positive targets, to continuously verify strain susceptibility profiles; (ii) a negative growth control comprising inoculated MHB treated with vehicle buffer alone to confirm log-phase replication; and (iii) a sterility control utilizing sterile, uninoculated MHB to guarantee medium sterility and establish spectrophotometric blank baselines. To ensure reproducibility, three independent biological replicates were performed at weekly intervals using freshly prepared bacterial suspensions.

### 2.8 Antimicrobial susceptibility testing

To evaluate the truncation effect of Lfcin B on structural-functional relationship, nine clinically relevant bacterial strains were selected: *E. coli* ATCC 25922, *K. pneumoniae* ATCC 4352, *P. aeruginosa* ATCC 27853, *S. typhimurium* ATCC 14028, *S. gallinarum* isolate, *S. flexneri* ATCC 12022, *S. aureus* ATCC 25923, *T. pyogenes* ATCC 19411, and *T. pyogenes* isolate. Antibacterial susceptibility was determined using standardized agar disk diffusion (ADD) assays. Briefly, pre-warmed MHA plates were inoculated with 100 μL aliquots of bacterial suspension (1 × 10^8^ CFU/mL) and overlaid with commercially prepared antibiotic discs. ADD assays employed a standardized peptide concentration (200 μg/disc) followed by 20 h incubation at 37 °C. Ciprofloxacin discs (1.25 μg/disc) served as positive controls across all strains, with sterile MHB-impregnated discs constituting negative controls. To ensure experimental reproducibility, all susceptibility tests were conducted in nine biological replicates per trial.

### 2.9 Statistical analysis

Quantitative results are presented as mean ± standard deviation. Between-group differences were assessed using a two-tailed Student’s t-test, with statistical significance defined as *p* < 0.05. All statistical analyses, including data visualization, were performed using R software (version 4.4.2; R Core Team, 2023) and Origin (version 2025, OriginLab).

## 3 Results

### 3.1 Peptide syntheses, purifications and identifications

**Figure 2:**
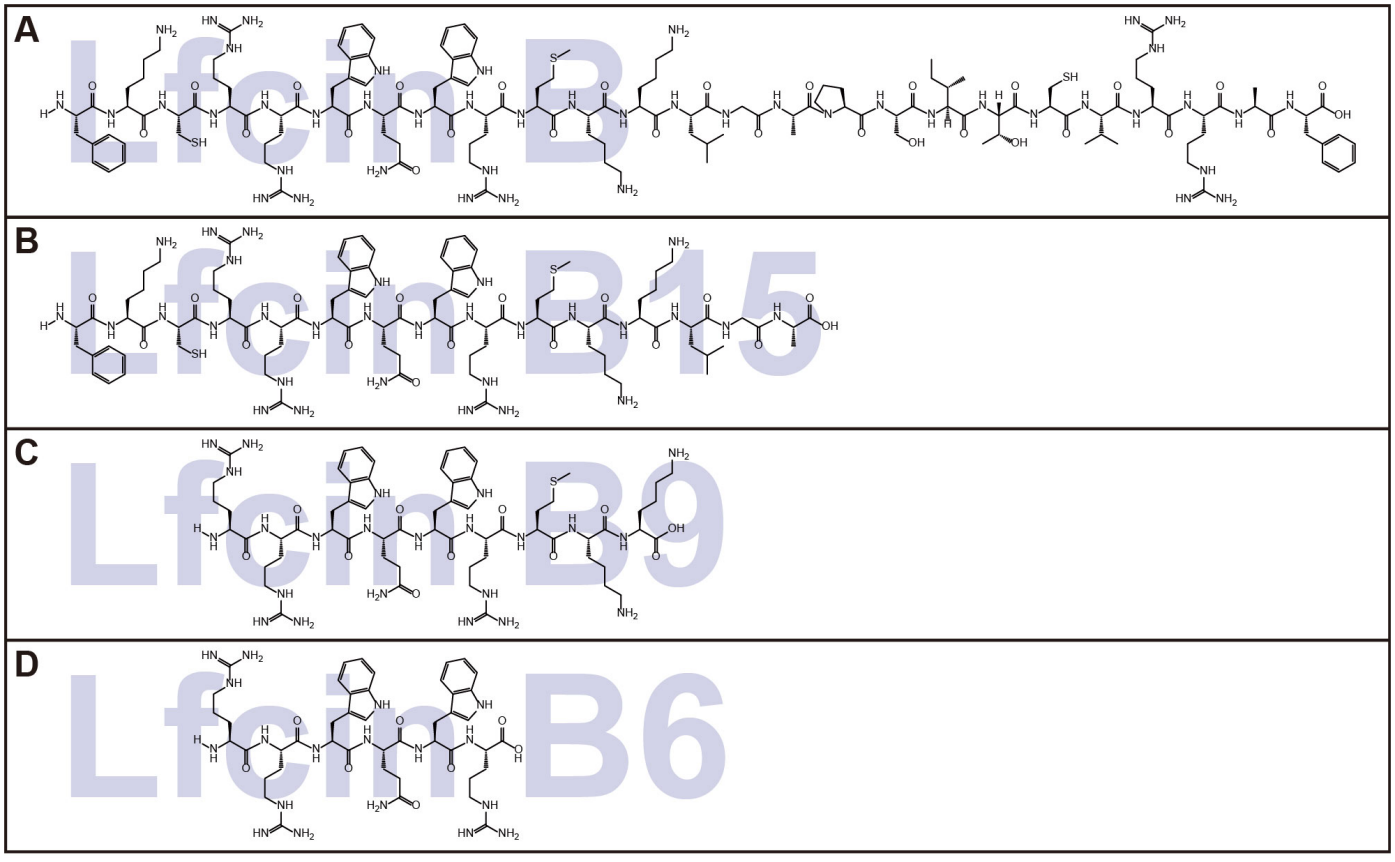
Molecular structures of Lfcin B and its truncated varians. The molecular structures of Lfcin B **(A)** and its truncated variants Lfcin B15 **(B)**, Lfcin B9 **(C)**, and Lfcin B6 **(D)** were aligned according to the shared amino acid residues.

Lfcin B6, B9, B15, and Lfcin B peptides were synthesized via SPPS. Purification using solid-phase extraction chromatography yielded high-quality peptides, with purities exceeding 95% as validated by RP-HPLC. Schematic representations of the peptide structures are presented in **Figure 1**. Molecular weights, confirmed by MALDI-TOF-MS, aligned closely with theoretical values. **Figure 2** depicts the molecular structures, while **Table 1** provides a comprehensive overview of all synthetic peptides utilized in this study.

**Table 1:** Synthetic peptides tested for antibacterial activity.

Peptides	Amino acid sequences (N terminal to C terminal)	Length (AA)^a^	Molecular weight	Purity	Net Charge	Percent of basic amino acid residues	Percent of hydrophobic amino acid residues	Hydrophobicity Index
Lfcin B6	RRWQWR	6	987.13	> 95%	3	50.00%	33.33%	-3.133
Lfcin B9	RRWQWRMKK	9	1374.68	> 95%	5	55.56%	33.33%	-2.744
Lfcin B15	FKCRRWQWRMKKLGA	15	1994.46	> 95%	6	40.00%	40.00%	-1.207
Lfcin	FKCRRWQWRMKKLGAPSITCVRRAF	25	3125.82	> 95%	8	32.00%	40.00%	-0.576

^a^ The abbreviation AA stands for amino acid.

### 3.2 Circular dichroism spectra

**Figure 3:**
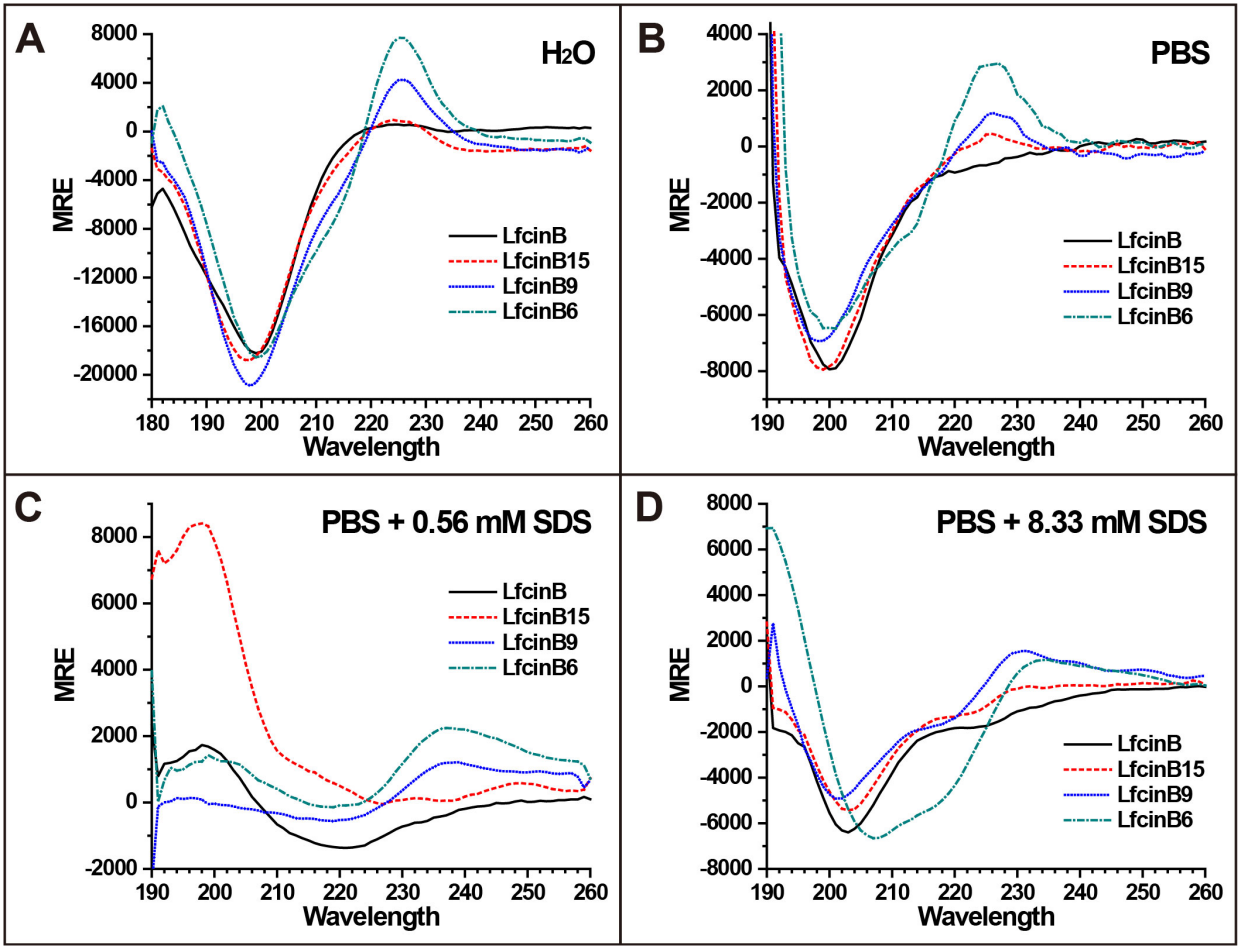
CD spectral analysis of Lfcin B and its truncated variants under distinct solution conditions. Peptides at a concentration of 0.16 mg/mL were analyzed in four environments: **(A)** deionized H_2_O, **(B)** PBS, **(C)** PBS with 0.56 mM SDS, and **(D)** PBS with 8.33 mM SDS. Spectra are color-coded to differentiate solution conditions, with the x-axis spanning the far-UV wavelength range (180–260 nm) and the y-axis representing mean residue ellipticity (*MRE*). SDS-containing conditions **(C, D)** simulate membrane-mimetic environments to probe structural changes in hydrophobic interactions.

In far-UV CD spectroscopy (180~260 nm), protein analysis typically requires sample concentrations between 0.1~0.5 mg/mL to achieve measurable signal-to-noise ratios. During empirical optimization trials with Lfcin B and its truncated variants, we identified 0.16 mg/mL as the optimal concentration for balancing spectral fidelity and baseline stability for these peptides. This concentration minimized buffer interference while maintaining sufficient peptide absorbance in the far-UV region, as confirmed by reproducible spectral features. **Figure 3** displays the CD spectra of the four synthesized peptides under varying conditions. In H_2_O, all peptides exhibit characteristic spectral curves with a prominent negative minimum at 198 nm and a positive maximum at 225 nm. At 198 nm, ellipticity values (in MRE) follow the sequence Lfcin B9 < Lfcin B15 < Lfcin B6 < Lfcin B, with Lfcin B6 exhibiting the strongest negative signal. Notably, Lfcin B6 demonstrates the highest ellipticity (most positive value) among the peptides (**Figure 3A**). In PBS, all peptides retain similar spectral profile comparable to that observed in H_2_O, maintaining a negative minimum at 200 nm and a positive maximum at approximately 226 nm (**Figure 3B**). But the ellipticity values for all peptides are significantly higher than those observed in H_2_O. When measured in PBS containing 0.56 mM SDS, the troughs observed in H_2_O and pure PBS invert into peaks for all peptides at 199 nm. Notably, Lfcin B15 exhibits the highest peak intensity among the four peptides (**Figure 3C**). At a higher SDS concentration (8.33 mM), all three peptides exhibited troughs at longer wavelengths (ranging from 202 nm to 207 nm) compared to those in the other solutions (**Figure 2D**). Notably, the spectral waveform of Lfcin B6 showed a more pronounced shift than those of the other peptides.

### 3.3 Secondary structure ratios

**Figure 4:**
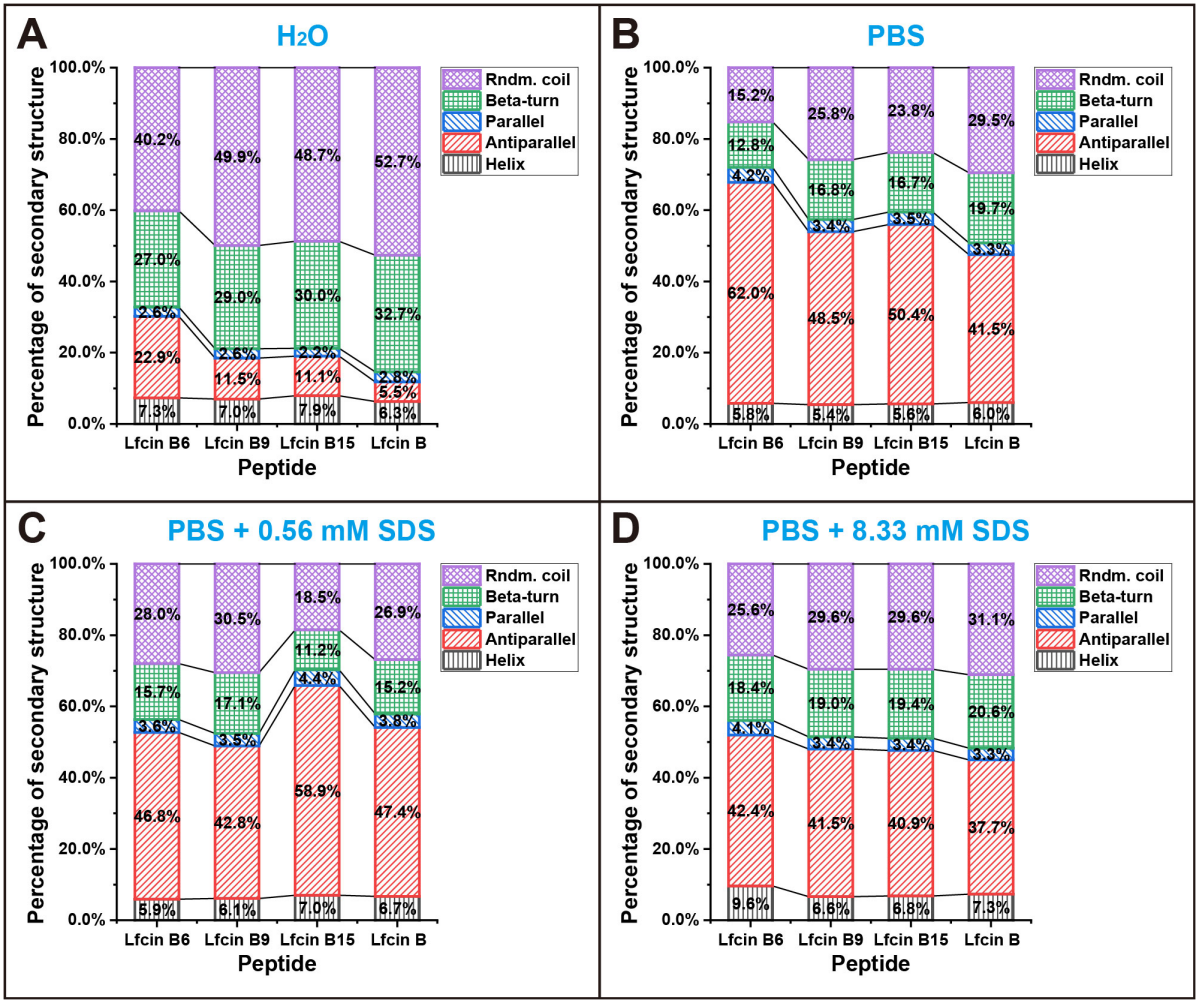
Quantitative analysis of secondary structure distributions for Lfcin B and its truncated variants under distinct solution conditions. Peptides were tested in four environments: **(A)** deionized H_2_O, **(B)** PBS, **(C)** PBS containing 0.56 mM SDS, and **(D)** PBS containing 8.33 mM SDS. Structural fractions (expressed as percentages) are categorized into five classes: random coil (Rndm. Coil), β-turn (Beta-turn), parallel β-sheet (Parallel), antiparallel β-sheet (Antiparallel), α-helix (Helix), and each represented by distinct colors. Secondary structure ratios were calculated via CD spectral deconvolution using neural network algorithms implemented in CDNN software (Applied Photophysics Ltd.).

**Figure 4** presents a quantitative analysis of the secondary structure composition for the four synthesized peptides. In H_2_O, all four peptides exhibit elevated proportions of random coil conformations, as demonstrated in **Figure 4A**. Specifically, Lfcin B, Lfcin B15, and Lfcin B9 display random coil contents of approximately 50%, whereas Lfcin B6 shows a notably lower ratio at 40.2%. Notably, Lfcin B6 exhibited a relatively stronger antiparallel β-sheet-like conformational tendency (22.9%) compared with the other peptides. In contrast, Lfcin B exhibits the lowest ratios of both α-helix (6.3%) and antiparallel β-sheet (5.5%) among the four analyzed peptides. When analyzed in PBS, the four peptides exhibited a pronounced shift toward ordered secondary structures, characterized by significantly reduced random coil content and a marked decrease in antiparallel β-sheet ratios compared to their structural profiles in H_2_O (**Figure 4B**). Notably, Lfcin B6 displayed a relatively stronger antiparallel β-sheet conformational tendency (62.0%) and a lower proportion of random coil components (15.2%) compared with the other peptides. In PBS containing 0.56 mM SDS, the peptides exhibit similar secondary structure ratios, with the exception of Lfcin B15 (**Figure 4C**). Lfcin B15 displays significantly higher proportions of antiparallel β-sheet (58.9%) and α-helix (7.0%) content compared to the other peptides. At an elevated SDS concentration (8.33 mM), Lfcin B9 and Lfcin B15 exhibit nearly identical ratios of secondary structures (**Figure 4D**). In contrast, Lfcin B6 demonstrates a higher α-helix (9.6%) and β-sheet (42.4%) content and reduced random coil conformation (25.6%) and β-turn (18.4%) structures, while Lfcin B displays lower antiparallel β-sheet content (37.7%) alongside elevated proportions of random coil (31.1%) and β-turn structures (20.6%).

### 3.4 Tertiary structure predictions

**Figure 5:**
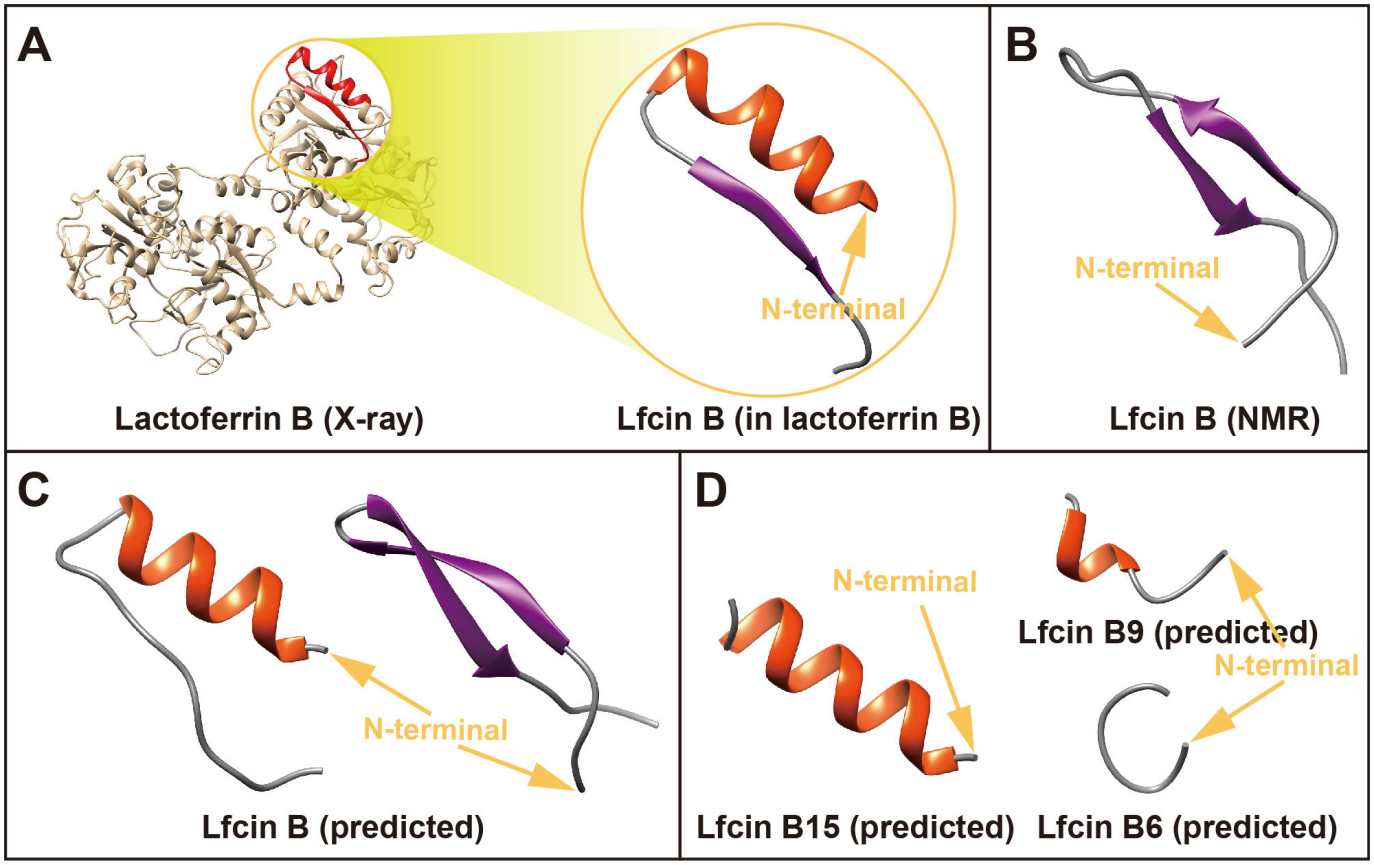
Tertiary structure analysis of Lfcin B and its truncated variants. **(A)** Lfcin B conformation within full-length lactoferrin, resolved by X-ray crystallography (PDB ID: 1BLF). **(B)** Solution-state structure of free Lfcin B determined by nuclear magnetic resonance spectroscopy (PDB ID: 1LFC). The peptide of C-terminal adopts an antiparallel β-sheet (blue) in aqueous environments, contrasting with the α-helical conformation observed in the intact protein. **(C)** In silico prediction of Lfcin B tertiary structure using AlphaFold3. **(D)** AlphaFold3-predicted structures of truncated variants Lfcin B15, B9, and B6. Structural orientations are indicated by golden arrows pointing to the N-termini. Secondary structure elements are color-coded: α-helices (red), β-sheets (blue).

AlphaFold3 predictions revealed distinct predicted structural models for Lfcin B, Lfcin B15, Lfcin B9, and the Lfcin B6 variant (**Figures 5C, D**). Notably, two distinct tertiary structures were predicted for Lfcin B: (1) An α-helix-rich conformer, characterized by a well-defined α-helix in the N-terminal half and a disordered C-terminal tail; (2) A β-sheet-dominant conformer, featuring antiparallel β-strands at both termini connected by a central flexible coil. Both conformers of Lfcin B exhibited 44.0% secondary structure content, with the α-helix-rich model comprising 11 residues in helical conformation and the β-sheet-dominant model containing 11 residues in β-strand configuration. For Lfcin B15, AlphaFold3 predicted a predominantly α-helical conformer spanning nearly the full length of the peptide (80.0% helical content). For Lfcin B9, the predictions indicated a tertiary structure with partial α-helical content (44.4%), though this helical propensity did not dominate the overall conformation. In contrast, the tertiary structure of Lfcin B6 was predominantly predicted to adopt a random coil state, lacking stable secondary structural elements.

The β-sheet-dominant conformer of Lfcin B exhibited high structural congruence with the NMR-derived solution structure of Lfcin B (PDB: 1LFC, RMSD = 1.3 Å), particularly in its antiparallel β-strand topology and flexible coil orientation (**Figure 5B**). In contrast, the α-helix-rich conformer of Lfcin B showed partial alignment with the X-ray crystallography structure of lactoferrin-embedded Lfcin B (PDB: 1BLF, RMSD = 2.8 Å) in helical registry. Notably, the full-length lactoferrin structure (1BLF) retains a C-terminal β-sheet motif absent in our isolated peptide predictions (**Figure 5A**). This discrepancy indicates that the β-sheet conformation observed in the embedded Lfcin B is stabilized by intermolecular interactions within intact lactoferrin, such as domain-domain contacts between the N-lobe and C-lobe.

The full-length Lfcin B sequence was explicitly integrated into the AlphaFold3 pipeline alongside its truncated derivatives to establish a standardized methodological benchmark. Cross-validating the full-length computational output against established experimental structures (PDB: 1LFC and 1BLF) allowed us to determine algorithmic reliability and alignment tolerances, thereby providing a validated baseline for interpreting the structural models of the truncated variants (Lfcin B15, B9, and B6) which lack empirical structural data.

### 3.5 Antibacterial activity

The synthetic peptides exhibited varying degrees of antibacterial activity against the tested bacterial strains, as summarized in **Table 2**. Full-length Lfcin B demonstrated superior antibacterial efficacy compared to its truncated variants (Lfcin B6, B9, and B15), with longer peptides generally exhibiting stronger antibacterial activity than shorter derivatives. Lfcin B6 showed limited activity, displaying antibacterial effects only against *T. pyogenes* ATCC 19411 and a clinical *T. pyogenes* isolate, both requiring an MBC of 250.0 μg/mL. Lfcin B9 exhibited a broader spectrum of activity than Lfcin B6, including *E. coli* ATCC 25922, a *S. gallinarum* isolate, *S. flexneri* ATCC 12022, *S. aureus* ATCC 25923, and both *T. pyogenes* strains. Notably, Lfcin B9 achieved an MIC_50_ of 62.5 μg/mL against *S. aureus* ATCC 25923. Lfcin B15 demonstrated antibacterial activity against all tested strains, though it remained less potent than full-length Lfcin B except against *S. aureus* ATCC 25923, where its efficacy surpassed that of the parent peptide. Full-length Lfcin B displayed the strongest overall activity, particularly against *T. pyogenes* ATCC 19411, with MIC_50_, MIC_90_, and MBC values of 3.9 μg/mL, 3.9 μg/mL, and 7.8 μg/mL, respectively. However, its activity against *S. aureus* ATCC 25923 was comparatively reduced, with Lfcin B9 and Lfcin B15 showing enhanced efficacy in this case.

**Table 2:** Antibacterial activity of the designed synthetic peptides against the four gram-negative bacteria.

Strain	Antibacterial index^a^	Lfcin B6^b^	Lfcin B9	Lfcin B15	Lfcin
*Escherichia coli* ATCC 25922	MIC_50_	> 1000.0 (1013.0)	500.0 (363.7)	125.0 (62.7)	62.5 (20.0)
	MIC_90_	> 1000.0 (1013.0)	500.0 (363.7)	250.0 (125.3)	62.5 (20.0)
	MBC	> 1000.0 (1013.0)	1000.0 (727.4)	250.0 (125.3)	62.5 (20.0)
*Klebsiella pneumoniae* ATCC 4352	MIC_50_	> 1000.0 (1013.0)	> 1000.0 (727.4)	1000.0 (501.4)	15.6 (5.0)
	MIC_90_	> 1000.0 (1013.0)	> 1000.0 (727.4)	> 1000.0 (501.4)	15.6 (5.0)
	MBC	> 1000.0 (1013.0)	> 1000.0 (727.4)	> 1000.0 (501.4)	15.6 (5.0)
*Pseudomonas aeruginosa* ATCC 27853	MIC_50_	> 1000.0 (1013.0)	> 1000.0 (727.4)	500.0 (250.7)	15.6 (5.0)
	MIC_90_	> 1000.0 (1013.0)	> 1000.0 (727.4)	1000.0 (501.4)	31.3 (10.0)
	MBC	> 1000.0 (1013.0)	> 1000.0 (727.4)	1000.0 (501.4)	31.3 (10.0)
*Salmonella typhimurium* ATCC 14028	MIC_50_	> 1000.0 (1013.0)	> 1000.0 (727.4)	62.5 (31.3)	31.3 (10.0)
	MIC_90_	> 1000.0 (1013.0)	> 1000.0 (727.4)	125.0 (62.7)	31.3 (10.0)
	MBC	> 1000.0 (1013.0)	> 1000.0 (727.4)	125.0 (62.7)	31.3 (10.0)
*Salmonella gallinarum* isolate	MIC_50_	> 1000.0 (1013.0)	500.0 (363.7)	62.5 (31.3)	31.3 (10.0)
	MIC_90_	> 1000.0 (1013.0)	500.0 (363.7)	62.5 (31.3)	31.3 (10.0)
	MBC	> 1000.0 (1013.0)	500.0 (363.7)	62.5 (31.3)	31.3 (10.0)
*Shigella flexneri* ATCC 12022	MIC_50_	> 1000.0 (1013.0)	1000.0 (727.4)	250.0 (125.3)	62.5 (20.0)
	MIC_90_	> 1000.0 (1013.0)	1000.0 (727.4)	> 1000.0 (501.4)	125.0 (40.0)
	MBC	> 1000.0 (1013.0)	1000.0 (727.4)	> 1000.0 (501.4)	125.0 (40.0)
*Staphylococcus aureus* ATCC 25923	MIC_50_	> 1000.0 (1013.0)	62.5 (45.5)	1000.0 (501.4)	> 1000.0 (319.9)
	MIC_90_	> 1000.0 (1013.0)	1000.0 (727.4)	> 1000.0 (501.4)	> 1000.0 (319.9)
	MBC	> 1000.0 (1013.0)	> 1000.0 (727.4)	> 1000.0 (501.4)	> 1000.0 (319.9)
*Trueperella pyogenes* ATCC 19411	MIC_50_	125.0 (126.6)	125.0 (90.9)	7.8 (3.9)	3.9 (1.2)
	MIC_90_	250.0 (253.3)	250.0 (181.9)	7.8 (3.9)	3.9 (1.2)
	MBC	250.0 (253.3)	250.0 (181.9)	15.6 (7.8)	7.8 (2.5)
*Trueperella pyogenes* isolate	MIC_50_	250.0 (253.3)	250.0 (181.9)	7.8 (3.9)	3.9 (1.2)
	MIC_90_	250.0 (253.3)	250.0 (181.9)	15.6 (7.8)	7.8 (2.5)
	MBC	250.0 (253.3)	250.0 (181.9)	15.6 (7.8)	7.8 (2.5)

^a^ The MIC_50_ and MIC_90_ refer to the concentrations needed to inhibit 50% and 90% of the strains, respectively; ^b^the MIC_50_, MIC_90_, and MBC values are expressed in μg/mL (μM).

### 3.6 Antimicrobial susceptibility

**Figure 6:**
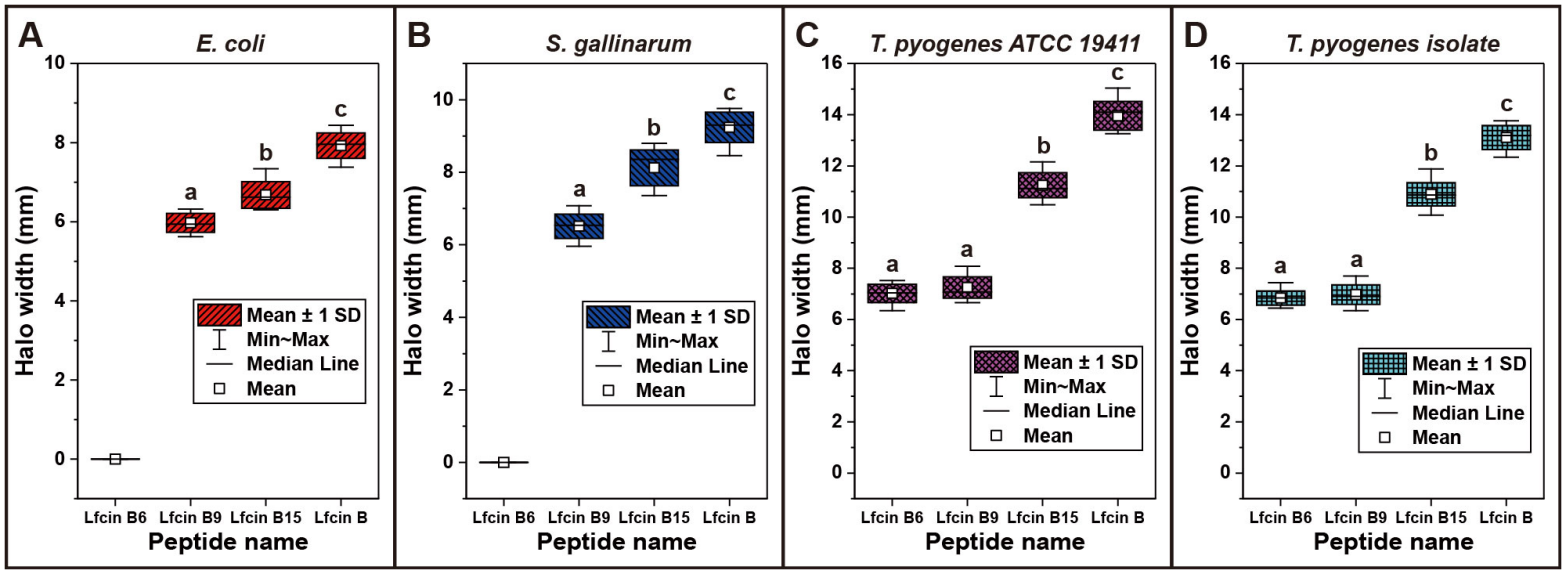
Antibacterial activity of Lfcin B and its variants assessed by disc diffusion assays. Inhibition zones for Lfcin B and truncated variants against **(A)** *Escherichia coli* ATCC 25922, **(B)** isolate of *Salmonella gallinarum*, **(C)** *T. pyogenes* ATCC 19411, and **(D)** isolate of *T. pyogenes* are exhibited in box plots. Sterile 6-mm discs impregnated with 20 μL peptide solution (20 mg/mL) were placed on Mueller-Hinton agar plates uniformly inoculated with 1 × 10^8^ CFU/mL bacterial suspensions. Plates were incubated at 37 °C for 20 h. Inhibition zone diameters (edge-to-edge, including disc) were measured directly; halo widths (distance from disc edge to growth boundary) are reported as (total zone diameter – disc diameter)/2. Halo width data are expressed as mean ± SD. Distinct lowercase letters above error bars indicate statistically significant differences between treatment groups.

In susceptibility assays (**Figure 6**), inhibition zone sizes demonstrated a clear length-dependent trend: Lfcin B exhibited the largest zones against all tested bacterial strains, significantly surpassing its truncated variants. Minimal activity was observed for Lfcin B6, which produced detectable zones only against the two *T. pyogenes* strains, consistent with its MIC/MBC values. Lfcin B9 showed broader activity, inhibiting most strains but remaining ineffective against *K. pneumoniae* ATCC 4352, *P. aeruginosa* ATCC 27853, and *S. typhimurium* ATCC 14028. Consistent with observations from MIC and MBC assays, inhibition zones progressively increased with peptide length across variants, culminating in the largest zone observed for full-length Lfcin B against *T. pyogenes* ATCC 19411.

## 4 Discussion

### 4.1 Salinity-driven structural reorganization and functional implications

The CD spectra of Lfcin B and its truncated variants reveal critical information about their solvent-dependent secondary structural configurations (**Figure 3**). The spectral profiles of Lfcin B in this study align closely with those reported previously under identical solvent conditions (Pei et al., 2021) and show consistency with independent findings, despite differences in experimental setups (Huertas Méndez et al., 2017), underscoring the robustness of the analytical methods employed for peptide structural characterization. In H_2_O, Lfcin B and its variants adopted predominantly random coil conformations (**Figure 4A**). Strikingly, under saline conditions, noticeable reductions occurred in random coil and β-turn content, with a concomitant increase in antiparallel β-sheets (**Figures 4B–D**). This shift suggests that physiological ionic conditions may contribute to a more ordered structural state in these peptides, potentially by stabilizing conformations that could be relevant to their biological activity. Notably, β-turn formation was most prominent in H_2_O, corroborating earlier reports that Lfcin17–30 adopts more defined β-turns in low-ionic-strength buffers compared to high-ionic-strength systems (Bolscher et al., 2009). Conversely, antiparallel β-sheet content remained significantly lower in H_2_O than in saline, indicating that this structural motif is preferentially stabilized under physiological ionic conditions, consistent with prior studies (Hwang et al., 1998).

### 4.2 Conformational shifts modulated by SDS concentration

The critical micelle concentration (CMC) represents the threshold at which surfactants such as SDS self-assemble into micellar structures in solution (Zhong et al., 2015). For SDS, this occurs at approximately 8 mM in aqueous environments, forming membrane-mimetic aggregates characterized by a hydrophobic core (composed of clustered alkyl chains) and a hydrophilic shell enriched with negatively charged sulfonic acid groups. These micelles structurally resemble the anionic surfaces of bacterial membranes, providing a model system to study peptide-membrane interactions. Lfcin B is known to engage with bacterial membranes via electrostatic interactions between its cationic residues and the negatively charged phospholipid head groups on microbial surfaces (Huertas Méndez et al., 2017). In our study, exposure to 8.33 mM SDS (above the CMC) induced notable conformational changes in Lfcin B and its variants (**Figure 4D**). Specifically, we observed a significant increase in the proportion of random coil, β-turn, and α-helical structures, alongside a reduction in antiparallel β-sheet content compared to PBS controls. This suggests that SDS micelles induce conformational rearrangements with an increased propensity for β-turn and α-helix formation, which may reflect peptide behavior in membrane-like hydrophobic and charged environments.

When SDS concentrations fall below the CMC, micelles dissociate into monomeric surfactant molecules, altering their mode of interaction with peptides (Lu et al., 2018). All peptides exhibited analogous structural change trends across solutions except in PBS containing 0.56 mM SDS (**Figure 4C**), where divergent behavior highlighted distinct adaptive responses to hydrophobic microenvironments (Yeaman and Yount, 2003). Such structural plasticity is essential for antimicrobial function, enabling dynamic reorientation of the peptides during membrane insertion and disruption—a hallmark mechanism of AMPs (Sani and Separovic, 2016; Chen et al., 2023). At this sub-CMC concentration, Lfcin B6 and Lfcin B9 exhibited enhanced random coil and β-turn content and reduced antiparallel β-sheet structures relative to PBS. In contrast, Lfcin B15 and full-length Lfcin B displayed decreased random coil and β-turn ratios, with a concomitant increase in antiparallel β-sheet formation. This divergence highlights a potential structural dichotomy: shorter Lfcin B fragments may adopt more flexible conformations in monomeric SDS environments, while longer variants exhibit a propensity to stabilize antiparallel β-sheets under hydrophobic conditions. Such differences could reflect variations in peptide hydrophobicity, charge distribution, or length-dependent folding dynamics.

The unique behavior of Lfcin B15 in submicellar (0.56 mM) SDS warrants detailed mechanistic consideration. Lfcin B15 retains a net positive charge of +6 and an optimal proportion of hydrophobic residues (~40%), resulting in a balanced amphipathic character that favors both electrostatic attraction and hydrophobic interactions. At submicellar SDS concentrations, where monomeric SDS molecules predominate, these surfactant monomers may interact with the clustered cationic residues of Lfcin B15 without the steric constraints associated with micellar assemblies (Arsenault et al., 2010). This interaction pattern may facilitate the nucleation of more ordered, membrane-adaptive conformations (Yeaman and Yount, 2003). More broadly, the distinct behavior of Lfcin B15 under submicellar SDS conditions likely reflects its intermediate position within the peptide length spectrum, which confers enhanced conformational adaptability compared with both shorter truncated variants and the full-length peptide (Zhao et al., 2025). In such a heterogeneous monomeric surfactant environment, peptide-SDS interactions are highly sequence-dependent, and Lfcin B15 may achieve an optimal balance between electrostatic attraction and hydrophobic driving forces, leading to its distinctive structural rearrangements relative to other variants.

The observed discrepancies between AlphaFold3 predictions and CD-derived secondary structure estimates are expected, as AlphaFold3 provides static, energetically favorable structural models (Abramson et al., 2024), whereas CD spectroscopy reflects the dynamic and solvent-dependent conformational ensemble of peptides in solution (Wärmländer et al., 2025). AlphaFold3 generates structure predictions based on learned sequence-structure relationships and physicochemical constraints, often yielding more ordered conformations with increased secondary structure content, such as a tendency toward α-helical regions in Lfcin B15. In contrast, under experimental aqueous conditions, solvent molecules compete for backbone hydrogen bonding, leading to more flexible and less ordered conformational states as captured by CD measurements. These differences highlight that computational predictions and experimental measurements provide complementary perspectives on peptide structure rather than identical structural descriptions.

### 4.3 Alterations in antibacterial activity

Standardization of bacterial inoculum density is essential for ensuring reproducibility and comparability in antimicrobial susceptibility testing. Method-dependent variations in MIC values have been reported for Lfcin B, highlighting the importance of consistent experimental conditions. In this study, all susceptibility assays were performed under standardized protocols following established guidelines (Wiegand et al., 2008), thereby minimizing methodological variability and ensuring reliable comparison across peptide variants and bacterial strains.

Our findings reveal that the MIC of Lfcin B against *E. coli* ATCC 25922 (62.5 μg/mL) (**Table 2**) aligns closely with previously reported values ranging from 30 to 100 μg/mL (Leon-Calvijo et al., 2015; Bruni et al., 2016; Huertas Méndez et al., 2017). This consistency underscores the robustness of the standardized approach adopted here. Notably, the MIC observed in our study matches earlier data generated using identical methodologies (Pei et al., 2021), further validating the critical role of protocol harmonization in cross-study comparisons.

Against *K. pneumoniae* ATCC 4352, *P. aeruginosa* ATCC 27853, and *S. flexneri* ATCC 12022, antibacterial activity is nearly exclusive to Lfcin B (**Table 2**), indicating that its intact amino acid sequence is critical for its antimicrobial function. The susceptibility of *K. pneumoniae* strains to Lfcin B exhibited significant strain-dependent variation. In our study, *K. pneumoniae* ATCC 4352 showed pronounced sensitivity to Lfcin B, with an MIC of 15.6 μg/mL (**Table 2**), consistent with the MIC value reported in our prior work for the same bacterial strain (Pei et al., 2025). Conversely, *K. pneumoniae* JCM-1662T displayed reduced susceptibility, requiring a substantially higher MIC for Lfcin B as documented in earlier studies (Bellamy et al., 1992). These observed differences likely reflect inherent variations between the tested strains. For instance, ATCC 4352 may possess unique membrane permeability characteristics or altered efflux pump activity compared to JCM-1662T, which could explain its heightened sensitivity.

In this study, Lfcin B exhibited an MIC of 15.6 μg/mL against *P. aeruginosa* ATCC 27853 (**Table 2**), a value that contrasts sharply with the previously reported MIC of 5 μg/mL for a distinct *P. aeruginosa* strain isolated in an independent study (Nawal Abd et al., 2021). This disparity likely reflects inherent differences in genomic or phenotypic characteristics between the strains analyzed. Notably, ATCC 27853 is a standardized reference strain routinely utilized for antimicrobial susceptibility testing and quality assurance, whereas the strain examined by Nawal Abd et al (Nawal Abd et al., 2021). may represent a clinical isolate with divergent resistance mechanisms or adaptive traits. To elucidate the molecular basis of Lfcin B resistance, future studies should conduct comparative genomic analyses of *P. aeruginosa* strains, particularly focusing on hypervirulent or multidrug-resistant clinical isolates, to determine specific genetic or structural factors influencing susceptibility.

The antibacterial efficacy of Lfcin B is fundamentally linked to its amphipathic structure, where spatially organized hydrophobic and cationic residues facilitate membrane insertion via electrostatic interactions with negatively charged bacterial membranes (Lei et al., 2021; Semeraro et al., 2022), ultimately inducing cell death (Sinha et al., 2013). This mechanism aligns with the “carpet model”, wherein peptides parallel to the membrane surface disrupt integrity upon reaching critical concentrations (Islam et al., 2023). Despite truncated variants retaining similar proportions of basic/hydrophobic residues (**Table 1**), Lfcin B exhibited significantly enhanced activity against all tested pathogens, evidenced by lower MICs/MBCs and larger inhibition zones (**Table 2**, **Figure 6**), compared to its truncated variants. This functional discrepancy is hypothesized to involve the absence of Cys20 in truncated peptides, which prevents the formation of the intramolecular disulfide bond. The disulfide bond is essential for the antibacterial function of Lfcin B, as established in prior studies (Pei et al., 2020, 2021, 2025). This covalent linkage critically governs peptide stability and bioactivity, since the addition or removal of cysteine residues which enable disulfide bond formation directly modulates structural flexibility and conformational stability by altering the potential for these bonds (Li et al., 2012). Specifically, the disulfide bond enhances three key functional properties, including conformational stability, membrane permeability, and target binding affinity, while simultaneously conferring resistance to proteolytic degradation (Markus et al., 2021).

The present findings highlight a length-dependent structure-activity relationship of bovine Lfcin B, although peptide length itself may not be the sole determinant of antibacterial potency. Progressive truncation altered the conformational landscape of Lfcin B and reduced its structural adaptability under different environmental conditions. The ultra-short fragments Lfcin B6 and Lfcin B9 exhibited a greater tendency toward disordered conformations, as indicated by CD spectroscopy and AlphaFold3 predictions, with limited formation of stable secondary structural motifs. This reduced structural organization may contribute to their weakened antibacterial activity, as reflected by the high MIC values observed against most tested pathogens (Jenssen et al., 2006). Their shortened sequences may impair the ability to maintain an appropriate balance between cationic residues and hydrophobic regions required for effective membrane interaction (Zhao et al., 2025). In contrast, full-length Lfcin B and Lfcin B15 retained greater conformational adaptability and displayed environment-dependent structural responses, which were associated with stronger antibacterial activity. These findings suggest that sequence length, structural flexibility, and amphipathic organization collectively contribute to the antibacterial function of Lfcin B variants (Fathi et al., 2024).

Interestingly, Lfcin B15 appeared to represent a structurally and functionally informative intermediate among the truncated variants. Compared with Lfcin B6 and Lfcin B9, Lfcin B15 retained stronger antibacterial activity, suggesting that the additional residues contribute to maintaining effective amphipathic organization and membrane interaction (Zhao et al., 2025). However, its activity remained lower than that of full-length Lfcin B, indicating that the complete sequence and disulfide bond-mediated structural stabilization are important for achieving maximal antibacterial potency (Pei et al., 2021).

The enhanced antibacterial activity of Lfcin B15 against *S. aureus* may arise from an optimized balance between electrostatic attraction, hydrophobic insertion, and conformational flexibility (Fathi et al., 2024). Unlike the shorter fragments (Lfcin B6 and Lfcin B9), Lfcin B15 contains additional residues that extend the amphipathic interface, providing sufficient cationic and hydrophobic domains for effective interaction with the bacterial membrane. Meanwhile, compared with the full-length Lfcin B, the shorter Lfcin B15 may possess increased conformational adaptability, allowing it to adopt membrane-interacting conformations more efficiently under specific membrane environments (Oliveira Júnior et al., 2025). The distinct response of *S. aureus* may also reflect differences in membrane composition between Gram-positive and Gram-negative bacteria (Cardoso et al., 2024). As a Gram-positive bacterium, *S. aureus* lacks an outer membrane barrier, enabling direct interaction between Lfcin B15 and the cytoplasmic membrane. Therefore, the intermediate length and structural flexibility of Lfcin B15 may provide an optimal configuration for membrane disruption in *S. aureus*.

### 4.4 Limitations

A limitation of AlphaFold3-based structural prediction should be noted. Although AlphaFold3 represents a state-of-the-art approach for protein structure modeling, its predictions are primarily optimized for obtaining stable structural representations and may have limitations when applied to short, intrinsically disordered, or amphipathic antimicrobial peptides. For these peptides, predicted structures should be interpreted as low-energy conformational states or representative snapshots within a dynamic conformational ensemble rather than definitive static tertiary structures. It must be emphasized that secondary-structure deconvolution values for short peptides should be interpreted as semi-quantitative structural propensities rather than absolute coordinates, given that deconvolution algorithms are inherently calibrated against large, globular reference protein databases. Therefore, the observed CD features may reflect transient conformational propensity or peptide aggregation rather than a defined secondary structure. Although SDS is widely used as a membrane-mimetic system, it cannot fully replicate the heterogeneity of bacterial membranes, including lipid composition, membrane asymmetry, and protein components. Therefore, SDS-induced conformational changes should be interpreted as indicative of membrane interaction propensity rather than direct *in vivo* structural behavior.

## 5 Conclusion

This study establishes that the potent, broad-spectrum antibacterial activity of Lfcin B against multidrug-resistant pathogens strongly correlates with its intrinsic conformational plasticity and structural stability, properties critically associated with its full-length sequence. Under ionic and hydrophobic gradients mimicking microbial membranes, Lfcin B exhibits distinct solvent-dependent structural states including α-helix-rich and β-sheet-dominant configurations, suggesting a high membrane interaction capacity. While truncated variants (Lfcin B15, B9, B6) retain limited structural adaptability and exhibit detectable activity against specific pathogens, progressive shortening disrupts conformational flexibility, destabilizes secondary structures, and impairs the synergistic action of essential cationic and hydrophobic residues, resulting in significantly attenuated efficacy or complete functional loss. Interestingly, by maintaining an optimized balance of charge and hydrophobicity, Lfcin B15 demonstrated highly adaptive structural transitions and retained potent antimicrobial efficacy, even surpassing the parent peptide against *S. aureus*. Consequently, Lfcin B as an evolutionarily refined scaffold with adaptive folding mechanisms and robust antimicrobial properties, represents the optimal foundation for engineering next-generation therapeutics targeting antibiotic-resistant infections.

## Data Availability

The original contributions presented in the study are included in the article/supplementary material. Further inquiries can be directed to the corresponding authors.
