# Supplementary material for: Conformational plasticity and truncational effects on bovine lactoferricin: structural determinants of enhanced antimicrobial activity
Source: Front Cell Infect Microbiol. 2026 Jul 10;16:1888171. doi: 10.3389/fcimb.2026.1888171 (PMC13395647; doi:10.3389/fcimb.2026.1888171)
Supplement: Supplementary file 1 [file Table1.docx]

Supplementary Material 1: HPLC of Lfcin B, Lfcin B15, Lfcin B9, and Lfcin B6

# Article Title

Conformational plasticity and truncational effects on bovine lactoferricin: structural determinants of enhanced antimicrobial activity

# Journal Name

Frontiers in Cellular and Infection Microbiology

# Author names

Jie Pei, Lin Xiong, Qianyun Ge, Xiaoyun Wu, Min Chu, Pengjia Bao, Xian Guo

# Affiliation

Key Laboratory of Yak Breeding in Gansu Province, Lanzhou Institute of Husbandry and Pharmaceutical Sciences, Chinese Academy of Agricultural Sciences, Lanzhou, Gansu, China; Key Laboratory of Animal Genetics and Breeding on Tibetan Plateau, Ministry of Agriculture and Rural Affairs, Lanzhou, Gansu, China

# E-mail address of the corresponding author

guoxian@caas.cn

HPLC Report

Product Name : Lfcin B6 RR-6

Instrument No. : 03019

Lot No. : P151123-LR488176

Column : 4.6*250mm, Kromasil C18 5um

Solvent A : 0.1% trifluoroacetic in 100% acetonitrile

Solvent B : 0.1% trifluoroacetic in 100% water

Gradient A B

0.01min 18% 82%

25.0min 43% 57%

25.1min 100% 0%

30.0min STOP

Flow rate : 1.0ml/min

Wavelength : 220nm

Volume : 20ul

──────────────────────────────

Rank Time Height Area Conc.

──────────────────────────────

1 10.967 213802 234818 1.263

2 11.091 1011790 18110648 97.44

3 11.433 85448 240599 1.295

──────────────────────────────

Total 100

HPLC Report

Product Name : Lfcin B9 RK-9

Instrument No. : 08003

Lot No. : P151123-LR488177

Column : 4.6*250mm, Boston Crest ODS

Solvent A : 0.1% trifluoroacetic in 100% acetonitrile

Solvent B : 0.1% trifluoroacetic in 100% water

Gradient A B

0.01min 18% 82%

25min 43% 57%

25.1min 100% 0%

30min STOP

Flow rate : 1.0ml/min

Wavelength : 220nm

Volume : 10ul

Peak No. Ret Time Height Area Conc.

1 5.755 2099.378 12292.124 0.2471

2 9.680 8994.828 78715.352 1.5826

3 9.848 509555.406 4767911.500 95.8633

4 9.848 10357.136 86905.797 1.7473

5 12.077 3584.920 27830.648 0.5596

100

HPLC Report

Product Name : Lfcin B15 FA-15

Instrument No. : 03019

Lot No. : P151123-LR488161

Column : 4.6*250mm, Kromasil C18 5um

Solvent A : 0.1% trifluoroacetic in 100% acetonitrile

Solvent B : 0.1% trifluoroacetic in 100% water

Gradient A B

0.01min 16% 84%

25.0min 41% 59%

25.1min 100% 0%

30.0min STOP

Flow rate : 1.0ml/min

Wavelength : 220nm

Volume : 20ul

────────────────────────────────

Rank Time Height Area Conc.

────────────────────────────────

1 7.242 16850 127379 1.658

2 15.404 12478 137396 1.789

3 15.686 773071 7323583 95.34

4 16.035 5647 32833 0.4274

5 17.343 8068 60432 0.7867

────────────────────────────────

Total 100

HPLC Report

Product Name : Lfcin B FF-25-1

Instrument No. : 03019

Lot No. : P151123-LR488174

Column : 4.6*250mm, Kromasil C18 5um

Solvent A : 0.1% trifluoroacetic in 100% acetonitrile

Solvent B : 0.1% trifluoroacetic in 100% water

Gradient A B

0.01min 22% 78%

25.0min 47% 53%

25.1min 100% 0%

30.0min STOP

Flow rate : 1.0ml/min

Wavelength : 220nm

Volume : 20ul

──────────────────────────────────────

Rank Time Height Area Conc.

──────────────────────────────────────

1 11.597 12452.989 126118.758 3.2887

2 11.748 20472.971 62723.043 1.6356

3 11.903 361771.313 3646018.500 95.0757

──────────────────────────────────────

Total 100
