# Supplementary material for: Conformational plasticity and truncational effects on bovine lactoferricin: structural determinants of enhanced antimicrobial activity
Source: Front Cell Infect Microbiol. 2026 Jul 10;16:1888171. doi: 10.3389/fcimb.2026.1888171 (PMC13395647; doi:10.3389/fcimb.2026.1888171)
Supplement: Supplementary file 2 [file Table2.docx]

Supplementary Material 2: MS of Lfcin B, Lfcin B15, Lfcin B9, and Lfcin B6

# Article Title

Conformational plasticity and truncational effects on bovine lactoferricin: structural determinants of enhanced antimicrobial activity

# Journal Name

Frontiers in Cellular and Infection Microbiology

# Author names

Jie Pei, Lin Xiong, Qianyun Ge, Xiaoyun Wu, Min Chu, Pengjia Bao, Xian Guo

# Affiliation

Key Laboratory of Yak Breeding in Gansu Province, Lanzhou Institute of Husbandry and Pharmaceutical Sciences, Chinese Academy of Agricultural Sciences, Lanzhou, Gansu, China; Key Laboratory of Animal Genetics and Breeding on Tibetan Plateau, Ministry of Agriculture and Rural Affairs, Lanzhou, Gansu, China

# E-mail address of the corresponding author

guoxian@caas.cn


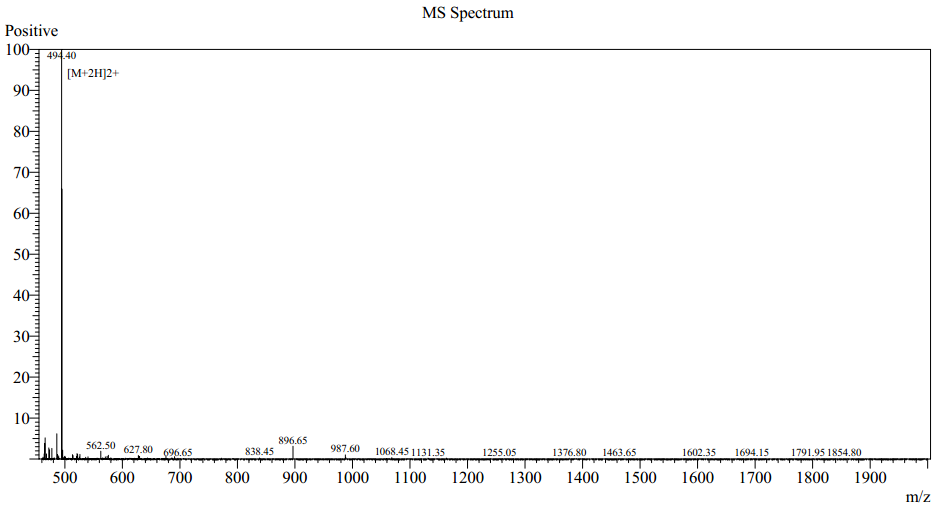


Probe : ESI Probe bias :+4.5kv

Nebulizer Gas Floe : 1.5L/min Detector :2.0kv

CDL : -20.0v T.Flow :0.2ml/min

CDL Temp : 250℃ B.conc :50%H2O/50%ACN

Block Temp : 200℃

Acquired by : Qiu

Data Acquired : 2024-10-21 11:55:24

Injection Volume : 1

Sample Name : LfcinB6 RR-6

Mw : 987.14

Lot No. : P151123-LR488176


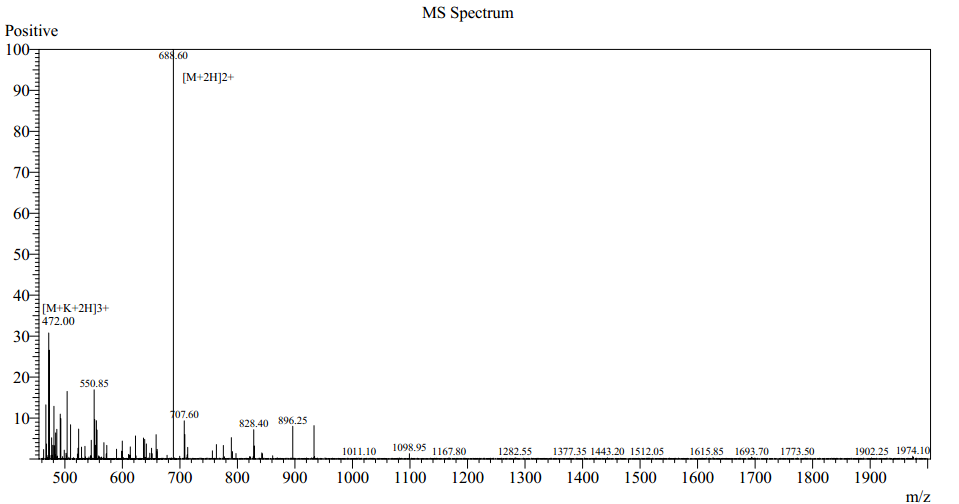


Probe : ESI Probe bias :+4.5kv

Nebulizer Gas Floe : 1.5L/min Detector :2.0kv

CDL : -20.0v T.Flow :0.2ml/min

CDL Temp : 250℃ B.conc :50%H2O/50%ACN

Block Temp : 200℃

Acquired by : Qiu

Data Acquired : 2024-10-21 16:09:39

Injection Volume : 1

Sample Name : Lfcin B9 RK-9

Mw : 1374.69

Lot No. : P151123-LR488177


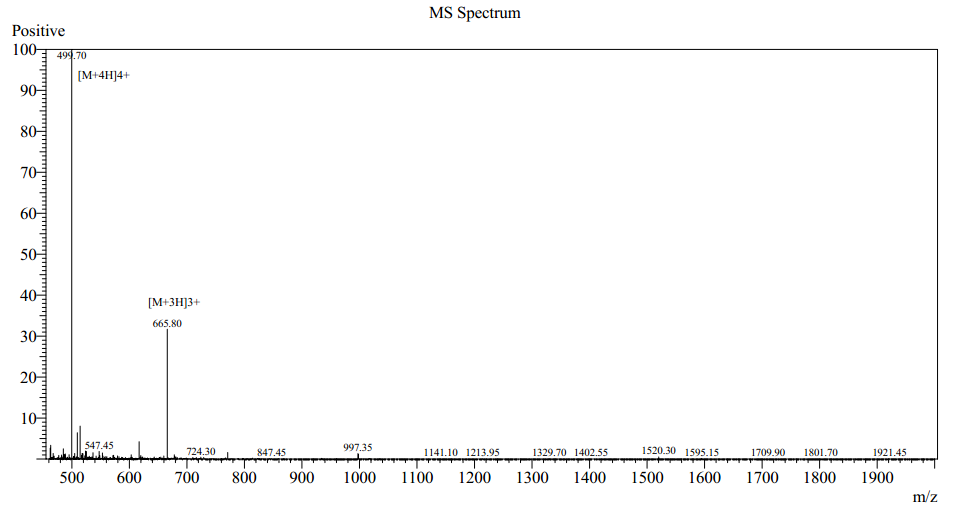


Probe : ESI Probe bias : +4.5kv

Nebulizer Gas Floe : 1.5L/min Detector :2.0kv

CDL : -20.0v T.Flow :0.2ml/min

CDL Temp : 250℃ B.conc :50%H2O/50%ACN

Block Temp : 200℃

Acquired by : Qiu

Data Acquired : 2024-10-21 11:55:24

Injection Volume : 1

Sample Name : Lfcin B15 FA-15

Mw : 1994.37

Lot No. : P151123-LR488161


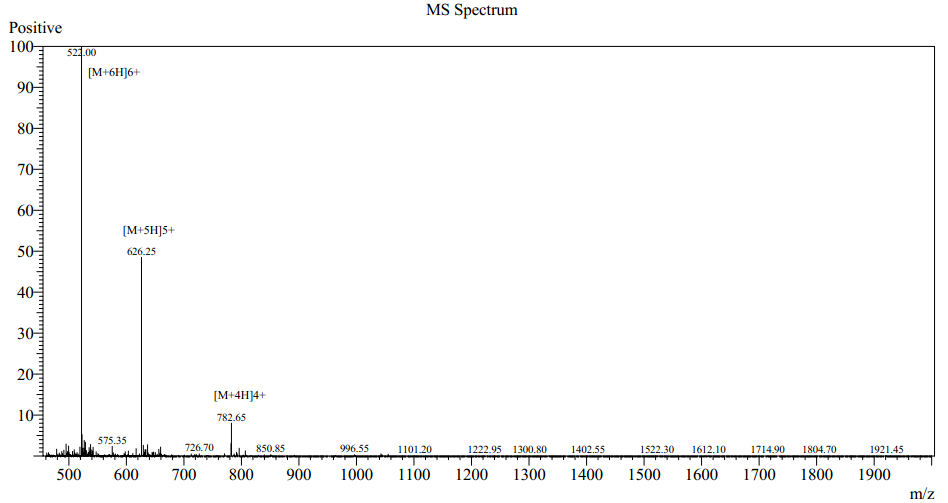


Probe : ESI Probe bias :+4.5kv

Nebulizer Gas Floe : 1.5L/min Detector :2.0kv

CDL : -20.0v T.Flow :0.2ml/min

CDL Temp : 250℃ B.conc :50%H2O/50%ACN

Block Temp : 200℃

Acquired by : Qiu

Data Acquired : 2024-10-21 11:55:24

Injection Volume : 1

Sample Name : Lfcin B FF-25-1

Mw : 3125.84

Lot No. : P151123-LR488174
